# Key inflammatory players for infarcted mass and cardiac remodeling after acute myocardial infarction

**DOI:** 10.3389/fcvm.2025.1609705

**Published:** 2025-07-18

**Authors:** Francisco A. H. Fonseca, Carolina N. França, Henrique A. R. Fonseca, Andrey J. Serra, Maria C. Izar

**Affiliations:** ^1^ Department of Medicine, Escola Paulista de Medicina da Universidade Federal de São Paulo, São Paulo, Brazil; ^2^ Post-Graduation Program in Health Sciences, Santo Amaro University (UNISA), São Paulo, Brazil; ^3^ Instituto Israelita de Ensino e Pesquisa, Albert Einstein Israelite Hospital, São Paulo, Brazil

**Keywords:** B cells, T cells, macrophages/monocytes, neutrophils, dendritic cells, cytokines, C-reactive protein, microbiota

## Abstract

Atherosclerosis has been defined as an inflammatory disease. As observed during acute infections, excess inflammatory activity is associated with disease severity and mortality. After myocardial infarction, several waves of inflammatory cells play a crucial role in infarct size and cardiac remodeling. In the short and long term, subtypes of inflammatory cells and cytokines released orchestrate the healing and stability of coronary disease. In recent years, some anti-inflammatory therapies have been shown to reduce the residual cardiovascular risk. Furthermore, some medications for treating risk factors and adoption of healthy lifestyle have decreased inflammatory markers and cardiovascular outcomes. In this complex network of possibilities, multiple interventions and not just on specific cell type or cytokine may provide better results. Finally, mild or moderate inflammatory activity appears necessary for better recovery and survival after acute myocardial infarction.

## Introduction

### Role of inflammation in main mechanisms of acute myocardial infarction

Despite continuous progress in therapeutic strategies, cardiovascular disease remains the leading cause of death worldwide (1). These deaths are mainly related to atherosclerosis, which can be defined as an inflammatory disease (2). Acute myocardial infarction with or without ST segment elevation are the most common thrombotic complications of coronary heart disease and are mainly related to plaque rupture or endothelial erosion, respectively (3). However, there are differences in the inflammatory mechanisms of these conditions in the set of acute coronary sindromes (3).

Vulnerable plaque is typically recognized in lesions characterized by a large lipid core within macrophages associated with apoptosis of these foam cells, forming debris in the intima. The imbalance between pro- and anti-inflammatory stimuli promoted by subtypes of lymphocytes and macrophages in the intima layer seems crucial to plaque rupture due to increased breakdown of matrix collagen (4). These proinflammatory stimuli are also related to thin fibrous cap. After rupture, the vulnerable plaque exposes highly thrombogenic constituents of plaque, leading to vessel occlusion (3, 4).

Plaque erosion has become increasingly common as a cause of acute coronary syndromes. Marked pathophysiological differences have been described between plaque erosion and plaque rupture. Eroded plaques usually occur in lipid poor plaques with increased matrix tissue (3). These plaques have increased content of proteoglycan and glycosaminoglycans (5, 6) and few inflammatory cells (7). Endothelial apoptosis may contribute to superficial erosion. Myeloperoxidase, a potent oxidant specie released by inflammatory cells, may promote endothelial death (8). More recently, the role of neutrophil extracellular traps was reported, showing endothelial cells activation and increased thrombogenicity through increased tissue factor expression (9).

### Cytokines and inflammatory cells in acute myocardial infarction

Acute myocardial infarction triggers waves of circulating inflammatory cells, in part beneficial but harmful when in excess (10). The first wave is characterized by the presence of polymorphonuclear neutrophils in the damaged myocardium. The second wave is dominated by the recruitment of macrophages that seem important for removal of cell debris contributing to myocardial healing (10). In parallel, there is an increased participation of lymphocytes, which may raise the presence of macrophages of pro-inflammatory phenotype (11). Alongside macrophages, there is an important participation of lymphocytes for changes in the phenotype of M1 pro-inflammatory macrophages into M2 macrophages and release of protective cytokines such as interleukins (IL) – 2, IL-4, and IL-10, involved in the myocardial repair (10). Conversely, the release of IL-6 in the first day of myocardial infarction seems related to increased infarcted mass, and reduced left ventricular ejection fraction, quantified by cardiac magnetic resonance imaging (12).

In patients with plaque erosion, the presence of neutrophil extracellular traps is associated with endothelium activation, promoting macrophage recruitment and increased thrombogenicity associated with augmented expression of IL-1α and interferon type 1 (IFN-1) (13). In addition, neutrophil extracellular traps can activate the NOD-,LRR-, and pyrin domain-containing protein (NLRP)3 inflammasome (14). Further, this inflammatory platform activates caspase 1, with subsequent release of IL-1β, and pro-IL-18, triggering the inflammatory pathway related to cardiovascular disease. Circulating IL-1β amplifies inflammatory and pro-thrombotic pathways due to increased expression of IL-6 and also due to its own expression by many inflammatory cells (15). Interestingly, the effects of NLRP3 inflammasome seem attenuated in the acute phase of myocardial infarction, modulated by enzymes released by monocytes, avoiding excessive inflammatory stimuli (16).

### Lymphocytes, monocytes, neutrophils, dendritic cells in acute myocardial infarction

**Figure 1:**
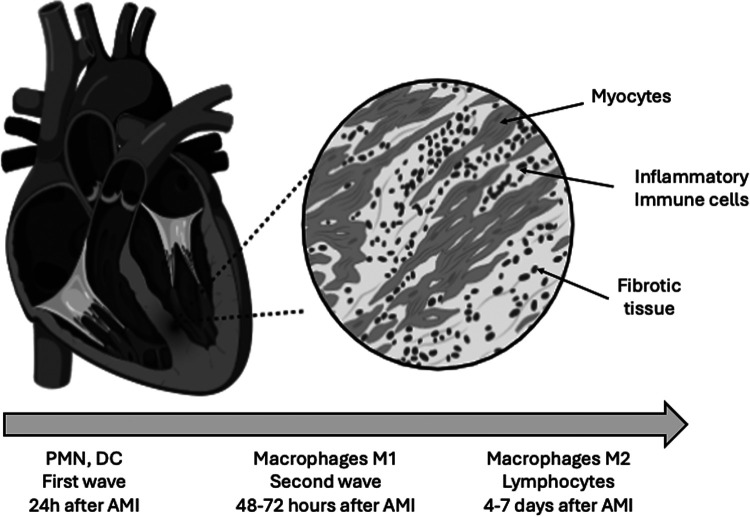
Waves of inflammatory cells after acute myocardial infarction. After coronary occlusion, the first wave of inflammatory cells are dominated by neutrophils (PMN) that can be seen in the first hours in the damaged myocardial. Dendritic cells (DC) are also early noted in the injured myocardial after reperfusion and these cells modulate the macrophage phenotype. The second wave is characterized by the presence of macrophages and, in parallel, lymphocytes. Macrophages are crucial for removal of cell debris. The lymphocytes are capable to modify the phenotype of macrophages into protective cells. Macrophages M2 are implicate in the release of anti-inflammatory cytokines involved in the myocardial healing (10–32).

The first stimulus for inflammatory cells recruitment is provided by necrotic myocytes with DNA fragments that act as danger-associated molecular patterns (DAMPs) (17). Next, the innate immune response is activated to clear cell debris from the region of myocardial infarction (18). The first mobilization of inflammatory cells is provided by neutrophils that are present at the myocardial infarct region in the first 24 h (18). In the same area, pro inflammatory monocytes and macrophages can be seen in the next 48–72 h, but are replaced by anti-inflammatory monocytes and macrophages for the days 4–7, which are important during the healing process of this phase (18) (Figure 1). Some studies conducted to evaluate early inhibition of neutrophils did not support protective effects in myocardial infarction size (19, 20). According to Nahrendorf (21), the initial infiltrate of monocytes are pro-inflammatory (M1) or C-C chemokine receptor type 2+(CCR2+), producing IL-1β and tumor necrosis factor alpha. Conversely, with the time, the monocyte phenotype that predominates is anti-inflammatory M2 or CCR2- (21, 22). Specimens obtained from patients who died in different post-infarction periods revealed a temporal accumulation of monocyte subsets. In the early inflammatory phase predominates classical monocytes CD14+CD16- in the infarct border region. In contrast, in the late proliferative phase after myocardial infarction, the monocytes subsets have comparable distribution. In the same study, a marked depletion of monocytes from spleen was described in the acute phase of myocardial infarction (23). These monocyte subsets were examined in patients with STEMI, stable coronary heart disease and healthy volunteers. Intermediate monocyte subset is considered the most inflammatory subtype and was associated with peak troponin and IL-6. Classical monocyte subset was also associated with IL-6. Some years after the publication of Nahrendorf (21), it was identified a third monocyte subtype and currently, the monocytes can be classified in three subsets, CD14++CD16- (classical monocytes), CD14++CD16+ (intermediate monocytes), and CD14+CD16++ (non-classical monocytes). Higher counts of intermediate monocytes appear to be related to more extensive myocardial infarction (24). Reduced CD16 expression in the first day was an independent predictor of higher left ventricular ejection fraction (25). On the other hand, late recruitment of CD16+ monocyte subset seems important for the myocardial repair (26). In elderly patients, an increase of CD14+CD16+ monocyte subset has been reported (27). This type of monocytes release pro-inflammatory cytokines, contributing for a chronic systemic inflammation in these patients (27). The persistence of pro-inflammatory subsets of monocytes was reported among STEMI patients examined at baseline, one month and six months, despite optimal medical therapy with statins, antiplatelet, betablockers, and renin angiotensin system blockers (28).

Circulating lymphocytes decrease 90 min after reperfusion and is associated with worse prognostic (29). This drop in B and T lymphocytes seem related to the presence of these cells in the injured myocardial (18). B2 lymphocytes appear to be protective, and 30 days after myocardial infarction in humans, there was an association between these classical B2 cells (B2 memory plus B2 naïve) with better left ventricular ejection fraction examined by cardiac magnetic resonance imaging (12). T regulatory cells also appear to be beneficial for the healing phenotype of monocytes/macrophages, in part due to higher expression of transforming growth factor-beta 1 (11).

Secondary lymphoid organs are reservoirs for a variety of inflammatory cells, including B and T lymphocytes, and dendritic cells. Inflammatory cells present in the peritoneal cavity or even in the pericardium can influence tissue repair after myocardial infarction (30) as well as in atherosclerosis (31).

Excessive pro-inflammatory responses after acute myocardial infarction contributes to adverse ventricular remodeling. The effects of regulatory B cells in heart failure were examined in an experimental myocardial infarction model. The authors reported that regulatory B cells decreased the CCR2 in monocytes, reducing the mobilization of inflammatory monocytes to the heart, decreasing fibrosis, and promoting better ventricular function (32). In humans, reduced circulating regulatory B cells were found among AMI patients compared with stable coronary artery disease patients (33).

Dendritic cells are antigen-presenting cells, with crucial role in adaptive and innate immunity. These cells are present shortly after reperfusion myocardial injury, and contribute for a better cardiac remodeling. Dendritic cells modulate the inflammatory responses decreasing pro-inflammatory monocytes/macrophages and their release of pro-inflammatory cytokines (34, 35). Table 1 summarizes the role of inflammatory cells.

**Table 1:** Monocyte and lymphocyte subsets and role in acute myocardial infarction.

Cell types	Role in AMI
CD14++CD16- monocytes	Classical
CD14++CD16+ monocytes	Intermediate
CD14+CD16++ monocytes	Non-classical
B2 (naïve plus memory) lymphocytes	Beneficial
Regulatory B lymphocytes	Beneficial
Regulatory T lymphocytes	Beneficial
Dendritic cells	Beneficial

Higher amounts of intermediate monocytes are related to more extensive myocardial infarction (24); B2 cells related to better left ventricular ejection fraction (12); Regulatory B cells related to smaller fibrosis and better left ventricular ejection fraction (32); Regulatory T cells beneficial for the healing of injured myocardial (11); Dendritic cells change monocyte phenotypes to less inflammatory cells.

### Microbiota and systemic inflammation in myocardial infarction

After myocardial infarction, an increase in intestinal permeability to bacteria products contributes to systemic inflammation and cardiac remodeling (36). Recently, bacteria translocation and lipopolysaccharides were associated with STEMI and poor prognosis (37, 38). Besides, dysbiosis and decrease in gut microcirculation after myocardial infarction seems related not only to systemic inflammation, but also to increased thrombus formation (39). Circulating lipopolysaccharides are increased in patients with myocardial infarction and are also present in coronary thrombi (37). The mechanism linking lipopolysaccharides to thrombus formation involves platelet activation by cathepsin G (37).

**Figure 2:**
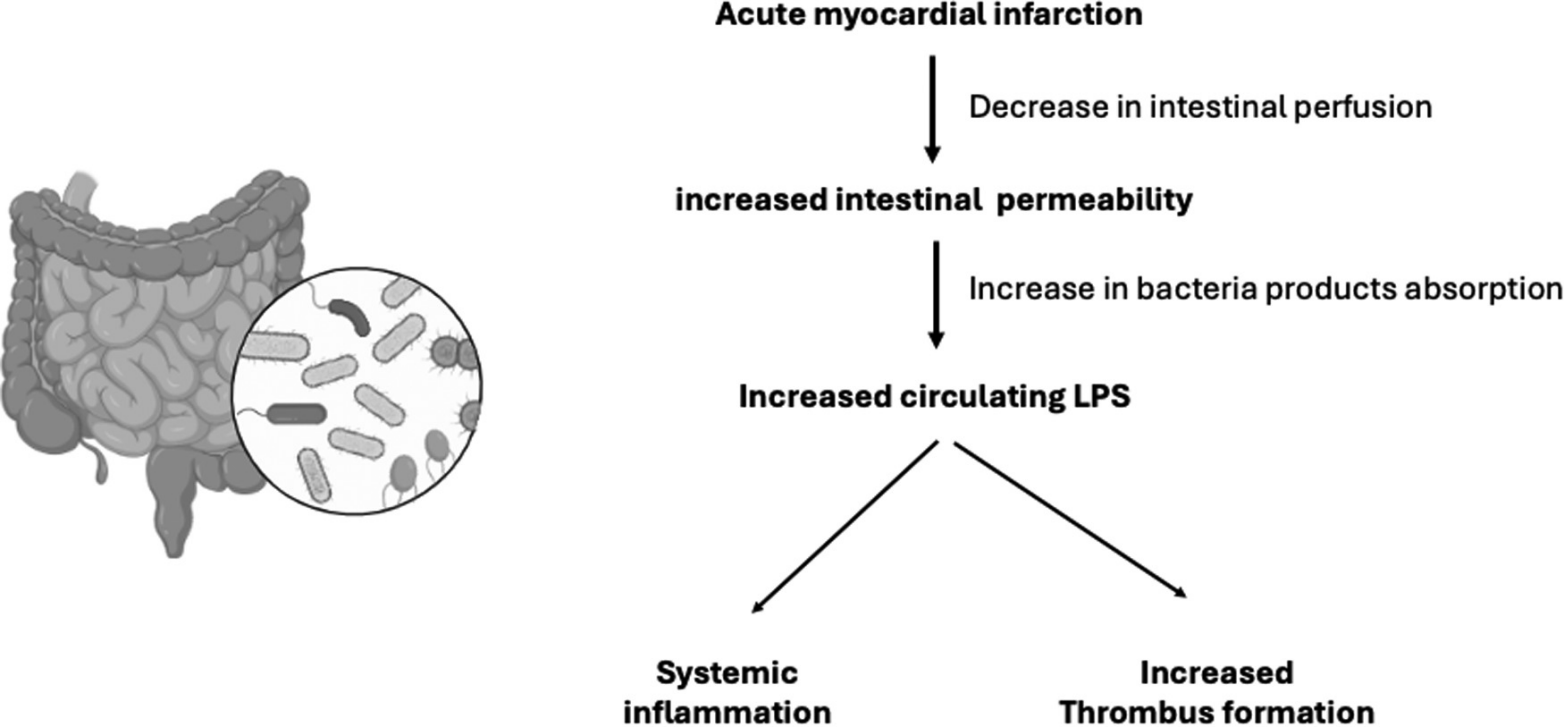
The heart-gut-microbiome-immune axis. After myocardial infarction, decrease in intestinal perfusion contributes to increase in intestinal permeability of bacteria products, including lipopolysaccharides (LPS). Increased LPS has been associated to poor ventricular function and increased infarction size. Circulating LPS is also related to increased thrombus formation (31–40).

The heart gut microbiome immune axis was examined in humans and by an experimental model of ischemia/reperfusion (40). Compared to healthy controls, patients with myocardial infarction had augmented circulating levels of markers of increased gut permeability such as lipopolysaccharides (40). The authors found that lipopolysaccharides positively correlated with myocardial infarct size and negatively with left ventricular ejection fraction (40). In the experimental model, an increased intestinal mucosa injury was observed following myocardial ischemia/reperfusion (40). Taken together, both studies reinforce the relevance of the heart gut microbiome immune axis (Figure 2).

### Role of the immune system in myocardial infarction

In patients with myocardial infarction, determinants of ventricular remodeling are not only related to early reperfusion, but also to the degree of inflammation and immune responses (41). The innate immune system was developed to ward off infections through a rapid protective response provided by a variety of inflammatory cells. However, following myocardial infarction, even in the absence of pathogens, the release of DAMPs by injured myocytes can activate the immune system (41). In the healthy myocardial, there are few resident mast cells, but after ischemia/reperfusion, these cells can release pro-inflammatory mediators capable to activate endothelium, monocytes/macrophages and neutrophils (42, 43). In fact, smaller infarct size was observed after ischemia/reperfusion, in mast cell deficient mice (44).

The innate immune responses after myocardial infarction can be activated by toll-like receptors and nucleotide-binding oligomerization domain-like receptors after recognition of DAMPs and inflammatory markers due to ischemia/reperfusion (41).

Several interventions on toll-like receptors (TLR2, TLR3, TLR4) have been examined in their role for cardiac remodeling after myocardial infarction (41). Among these toll-like receptors, the TLR4 antagonist eritoran revealed promising results (45).

**Figure 3:**
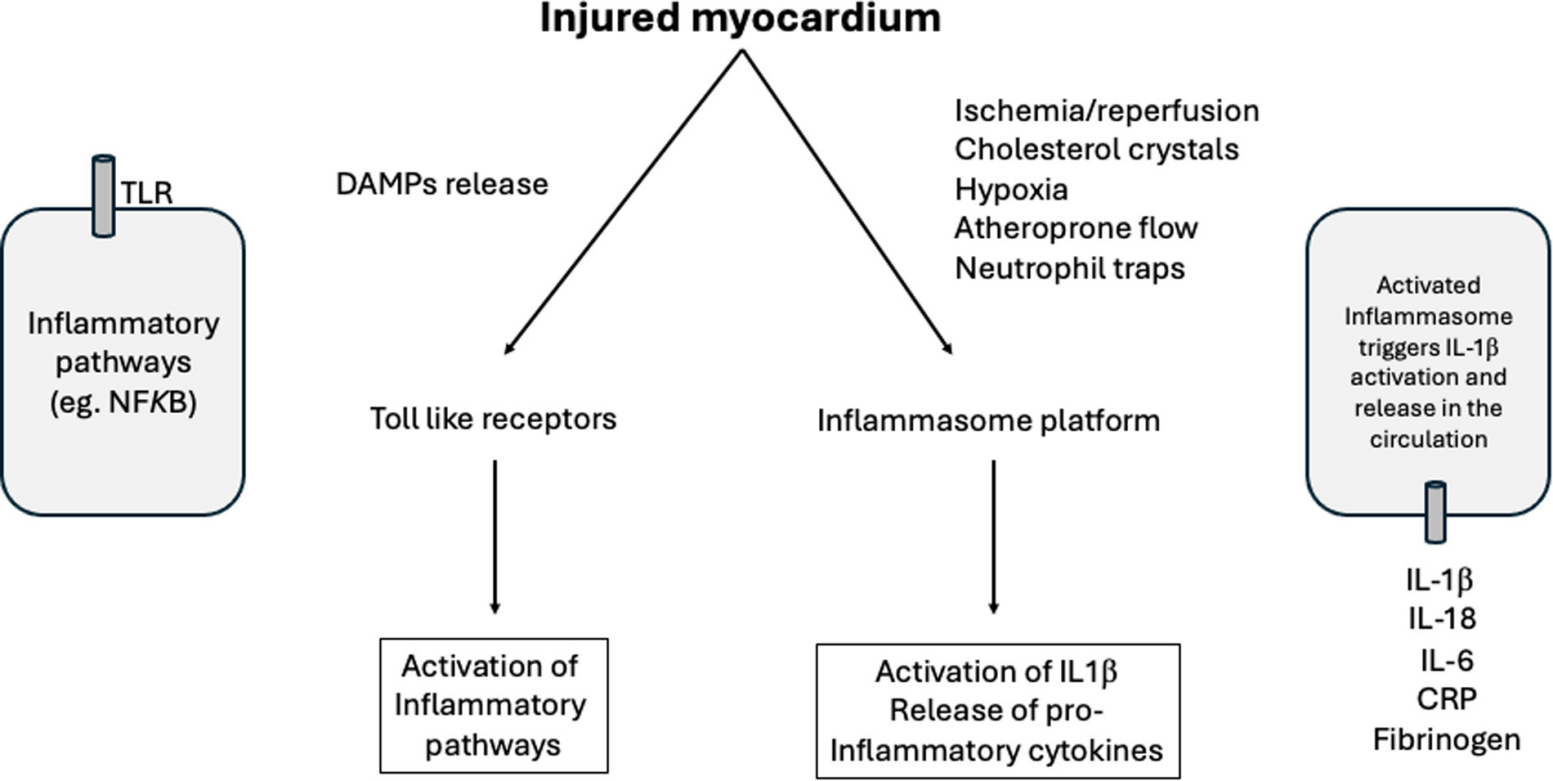
Role of the immune system in myocardial infarction. After ischemia/reperfusion there is a release from the injured myocardial of DAMPs that activate the immune system. The amount of resident mast cells are implicated in the degree of immune and inflammatory responses. After the release of DAMPs, the innate immune responses can be activated by toll-like receptors (TLR) and by the inflammasome platform. Several stimuli can trigger the activation of inflammasome, including ischemia/reperfusion, cholesterol crystals, neutrophil extracellular traps, atheroprone flow and hypoxia with subsequent release of pro-inflammatory cytokines. DAMPs, damage-associated molecular patterns; IL, interleukin; CRP, C-reactive protein; NFKB, Nuclear factor *kappa* beta (41–54).

The innate immune system can also be activated by the inflammasome platform (NLRP3). Several stimuli, including ischemia/reperfusion, activates NOD-like receptors promoting the release of IL-1β and IL-18 (15, 46). Once in the circulation, IL-1β interacts with inflammatory cells increasing the expression of IL-6 (15, 46). In CANTOS trial (47), the monoclonal human antibody canakinumab decreased high-sensitivity C-reactive protein (hsCRP) and IL-6 levels and main cardiovascular events, in the long term after myocardial infarction (48, 49). Inhibition of NLRP3 is an interesting target and may be associated with smaller myocardial infarct size (50–53). Common cardiovascular risk factors have been associated with inflammasome activation, including traditional risk factors linked to atherosclerosis. In this scenario, cholesterol crystals, ischemia/reperfusion, neutrophil extracellular traps, atheroprone flow, and local hypoxia are capable to activate inflammasome triggering the inflammatory cascade mediated by IL-1β and IL-6 (54) (Figure 3).

### How to estimate systemic inflammation?

Despite being a non-specific marker of inflammation, hsCRP is a very useful marker for cardiovascular risk stratification and for monitoring the treatment of cardiovascular disease (55). Plasma CRP is produced by the liver under transcriptional control by IL-6 (55). In patients with acute myocardial infarction, increase in hsCRP levels in the first 24 h was correlated with microvascular infarction estimated by cardiac magnetic resonance (56).

After myocardial infarction, hsCRP levels also predict adverse ventricular remodeling (12, 57–59). Recently, a large primary prevention population followed over a period of 20 years in the EPIC-NORFOLK cohort confirmed the independent association of hsCRP with major adverse cardiovascular events (60). In other large cohort involving US women, hsCRP was also independent predictor of major adverse cardiovascular events in a 30-year follow-up (61).

Interleukin 6 is also a strong marker for future myocardial infarction, supporting an important role of this cytokine in the complications of coronary atherosclerosis (62). In the CANTOS trial, decrease in IL-6 levels by canakinumab was associated with reduction in major cardiovascular events (49).

The effects of IL-6 receptor inhibition was also examined in the setting of acute myocardial infarction. Patients were randomized 1:1 to receive tocilizumab or matching placebo during percutaneous coronary intervention and the myocardial salvage index was quantified by cardiac magnetic resonance imaging 3–7 days after intervention (63). An increase in the myocardial salvage index (primary objective) was observed in the tocilizumab arm (63).

### Inflammation detected by magnetic resonance imaging

In patients with acute myocardial infarction, impaired right ventricular ejection fraction and higher NT-proBNP values were related by T1 mapping by hepatic magnetic resonance, an useful biomarker of cardio-hepatic axis to be explored in the setting of inflammation (64). Myocardial edema in non-injured tissue after myocardial infarction may indicate inflammation and adverse outcomes. On this regard, cardiac magnetic resonance performed in patients with myocardial infarction showed that higher T2 mapping values in non-infarcted myocardial or surrounding tissue were related to larger infarct size, microvascular obstruction, left ventricular dysfunction, and adverse cardiovascular outcomes (65).

### Role of adipokines in acute myocardial infarction

The role of adipokines in myocardial infarction has been reviewed. In the Copenhagen General Population Study (66), adiponectin was measured in 30.034 individuals. This observational study revealed that elevated plasma adiponectin was associated with heart failure, atrial fibrillation, aortic valve stenosis, and myocardial infarction. In the same study, genetic analysis did not show causality (66). There are pro-inflammatory adipokines beyond adiponectin, such as visfatin and resistin and anti-inflammatory adipokines as omentin and ghrelin, and some of uncertain effects such as leptin or apelin (67). Therefore, as in the case of cytokines, the imbalance of pro- and anti-inflammatory adipokines may affect the occurrence of myocardial infarction and its evolution.

### New perspectives for inflammation control and cardiovascular outcomes

A comprehensive review of major findings from several anti-inflammatory clinical trials has already been reported (68). In our review we chose some of the most relevant to clinical practice or those that served as proof of concept.

In CANTOS trial, involving patients with previous myocardial infarction, baseline hsCRP levels were predictors of hospitalization due to heart failure (69). Treatment with canakinumab not only decreased atherothrombotic events, but also rates of hospitalization due to heart failure (69). However, neutropenia was more common among patients treated with canakinumab than those assigned to placebo, and more deaths were attributed to infections in patients treated with the canakinumab pooled groups (incidence rate, 0.31 vs. 0.18 per 100 person-years) (47).

Ziltivekimab is a fully human monoclonal antibody against IL-6 ligand. In the RESCUE-2 trial, involving high risk patients with chronic kidney disease (stages 3–5) also presenting hsCRP levels ≥2 mg/L, a substantial decrease in hsCRP levels (> 90%) was found (68). In a similar study, the RESCUE trial, a comparable decrease in hsCRP was reported (71). In both studies the treatment was well tolerated and additional benefits were described, such as decrease in fibrinogen, serum amyloid A, haptoglobin, phospholipase A2, and lipoprotein (a) (70, 71). Based on these findings, large outcome studies are currently ongoing. The ZEUS trial enrolling patients with established atherosclerotic disease, chronic kidney disease (stages 3–4) and elevated hsCRP levels, aims to evaluate the effects of ziltivekimab compared to placebo in major cardiovascular and renal outcomes (72). Ziltivekimab is also currently tested among patients with preserved or moderately decreased left ventricular ejection fraction in the HERMES trial (73). In addition to these studies, in patients with acute myocardial infarction, early therapy with ziltivekimab is being tested in the ARTEMIS trial (NCT06118281).

Colchicine is an inexpensive anti-inflammatory drug that has been tested in patients with chronic and acute coronary disease. In the setting of acute myocardial infarction, the use of colchicine (0.5 mg once daily) or placebo, started within the first 30 days was examined in 4,745 patients with a median follow-up of 22.6 months (74). Those assigned to colchicine had 23% relative risk reduction on major cardiovascular events. The drug was well tolerated, but a modest increase in pneumonia rate was reported (74). More recently, a new large trial with a 2-by-2 factorial design, in patients with myocardial infarction, tested the effects of colchicine or placebo and spironolactone or placebo (75). In the trial, treatment with these drugs started soon after myocardial infarction, but neither spironolactone nor colchicine reduced major cardiovascular events (76, 77). Thus, after the CLEAR SYNERGY (OASIS 9) trial results, the effects of colchicine in major cardiovascular events after AMI seem controversial (76, 77) (Table 2).

**Table 2:** Cardiovascular outcomes in clinical trials with anti-inflammatory therapy.

Trial	Therapy	Main results	References
CANTOS	Three doses of Canakinumab (monoclonal antibody against IL-1beta) vs. placebo, median follow-up of 3.7 years	Decrease CV death, non-fatal MI, non-fatal stroke (primary objective) and secondary end point including hospitalization for UA leading to urgent revascularization	(58, 66)
COLCOT	Colchicine 0.5 mg once daily vs. placebo, median follow-up of 22.6 months	Decrease in 23% CV death, resuscitated cardiac arrest, myocardial infarction, stroke, urgent hospitalization due to UA leading to coronary revascularization (primary objective)	(71)
CLEAR SYNERGY	2 by 2 factorial design including spironolactone vs. placebo and either colchicine vs. placebo, median follow-up of 3 years	Negative results for the primary objective (CV death, recurrent MI, stroke, unplanned ischemia-driven revascularization)	(75–77)
RESCUE and RESCUE-2	Ziltivekimab (monoclonal antibody against IL-6 ligand), three doses vs. placebo, every 4 weeks, follow-up 24 weeks	Decrease in C-reactive protein, fibrinogen, serum amyloid A, haptoglobin, phospholipase A2, lipoprotein(a)	(67, 68)

CV, cardiovascular; MI, myocardial infarction; UA, unstable angina.

## Vaccines and immune therapies

Notably, the incidence of recurrent CV events is disproportionately higher within the first 30 days post-acute coronary syndrome compared to the long-term period, highlighting a critical window of vulnerability (78). Influenza and pneumococcal vaccines are associated with decrease in the risk of cardiovascular disease (79). Influenza infection has been identified as a potential catalyst for systemic inflammation and plaque destabilization, particularly during seasonal outbreaks. The virus may act as an external trigger that exacerbates the inflammatory milieu associated with unstable atherosclerotic lesions, thereby elevating the risk of both cardiovascular and cerebrovascular events during the influenza season (80). Particularly in the elderly, influenza vaccination is related to lower rates of acute coronary syndromes and stroke, or new cardiovascular events (80–82). The mechanism of cardiovascular protection after influenza vaccination seems related to decrease in plaque rupture and in the prothrombotic stimuli (83, 84). The hypothesis that preventing influenza infection during or shortly after an acute myocardial infarction (AMI) may reduce subsequent cardiovascular events was prospectively evaluated in the Influenza Vaccination After Myocardial Infarction (IAMI) trial, conducted across Scandinavian countries. This multicenter, double-blind, placebo-controlled trial investigated the in-hospital administration of a standard-dose (15 μg per strain) quadrivalent influenza vaccine vs. placebo in patients with acute myocardial infarction who were eligible for percutaneous coronary intervention (85). The trial was designed to test whether influenza vaccination, administered during the peak of immune activation—within 72 h of coronary angiography—could reduce major adverse cardiovascular events over 12 months. Findings from the IAMI trial were promising. Influenza vaccination, compared to placebo, was associated with a 28% relative reduction in the composite primary endpoint of all-cause mortality, recurrent myocardial infarction, or stent thrombosis [hazard ratio 0·72 (95% CI 0·52–0·99)]. Meta-analysis comprising six randomized controlled trials (RCTs) evaluated the impact of influenza vaccination compared with placebo in patients at high cardiovascular risk, encompassing a total of 6,735 participants (mean age 67 years; 51.3% women; 36.2% with established cardiac disease). The analysis demonstrated an association between influenza vaccination and a lower incidence of subsequent cardiovascular events, with a more pronounced effect observed in patients with recent acute coronary syndrome (86). In the context of patients with acute coronary syndromes, a large, multicenter, randomized study, evaluated the effects of double-dose influenza vaccine vs. standard dose, started during the first week after the coronary acute event. The patients were followed up to 12 months but no differences in cardiopulmonary outcomes were observed between groups (87). Therefore, the strategy of doubling the dose of influenza seems insufficient to enhance cardiopulmonary protection. These findings are in agreement with other large study showing neutral effects of the high-dose trivalent influenza vaccine compared with standard dose of quadrivalent influenza vaccine for mortality or cardiopulmonary hospitalization (88). A recent updated meta-analysis, incorporating the most recently published randomized trials, demonstrated that influenza vaccination is associated with a 34% reduction in the risk of major cardiovascular events compared to placebo or standard care. This protective effect was particularly pronounced in patients with recent acute coronary syndrome, among whom vaccination conferred a 45% lower risk of cardiovascular events within 12 months post-vaccination (89).

**Figure 4:**
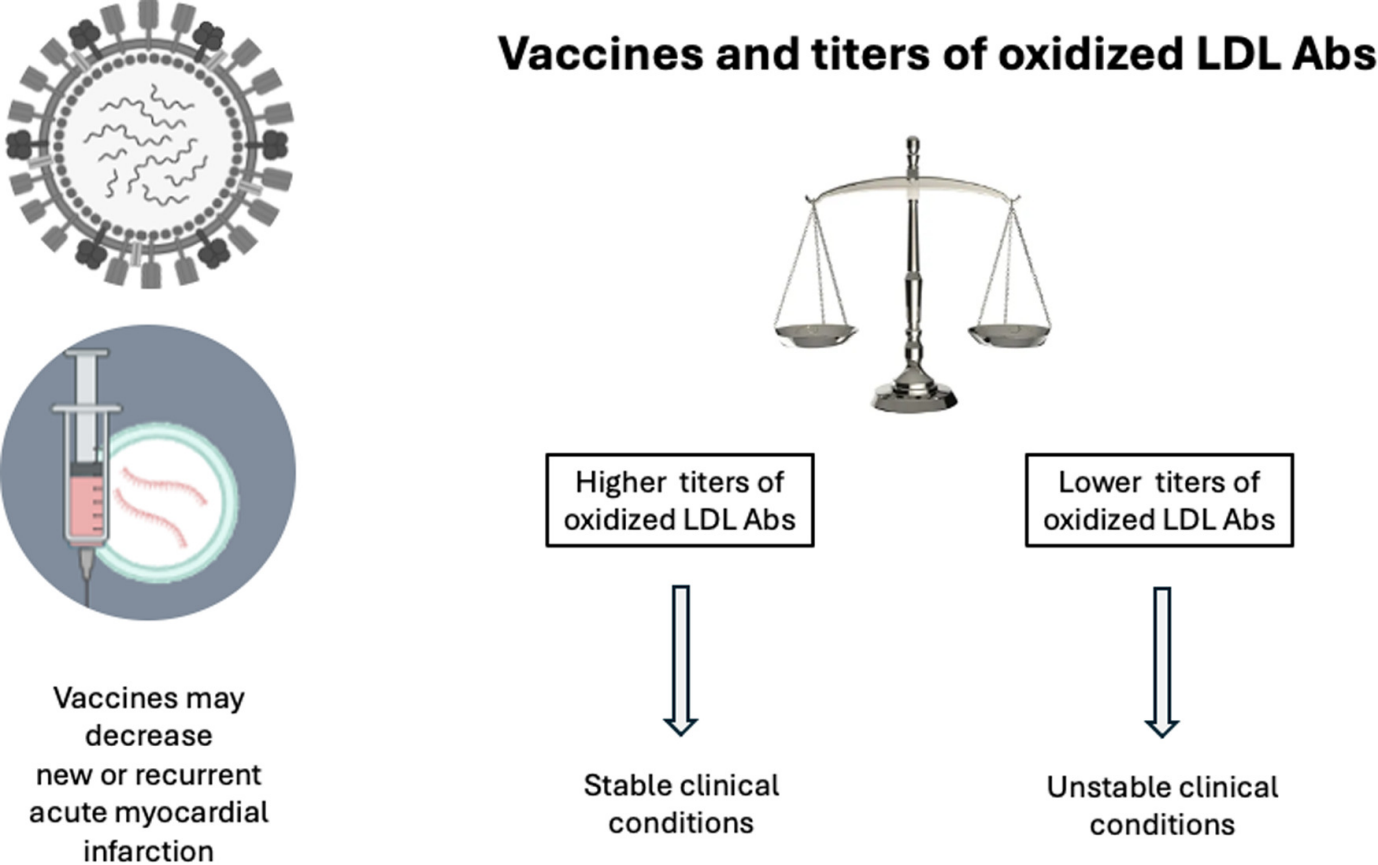
Vaccines and oxidized LDL Abs. Vaccines reduce the incidence of myocardial infarction due to decrease in inflammatory stimuli for plaque rupture and thrombosis. Uncontrolled cardiovascular risk factors seem related to decreased titers of oxidized LDL Abs. Conversely, stable clinical conditions are associated with higher titers of oxidized LDL Abs. Vaccination based on apo B epitopes have shown promising initial studies. Abs, antibodies (73–85).

Obesity and hypertension are common cardiovascular risk factors that are also associated with interesting differences in the immune responses against oxidized LDL. Among hypertensive patients, body mass index (BMI) and abdominal circumference were inversely related to the antibodies (Abs) anti oxidized LDL (90). Systolic and diastolic blood pressure were also inversely related to the titers of oxidized LDL-Abs and increased titers of inflammatory cytokines (91). Furthermore, treatment of hypertension increased the titers of oxidized LDL-Abs (89). In fact, high titers of autoantibodies against oxidized LDL appear to be a health marker, as suggested by the findings of their elevated titers in stable clinical cardiovascular conditions and lower titers in unstable patients (92). In this scenario of immune strategies, vaccination based on epitopes of apoB has been investigated, and promising anti-atherosclerotic results have been reported experimentally, suggesting a protective role mediated by regulatory T cells (93, 94) (Figure 4).

## Inflammation and perspectives with photobiomodulation

The possibility of modulating inflammatory responses after myocardial infarction has also been described in experimental model using photobiomodulation by laser. The authors reported transcriptional and post-transcriptional changes that can modify ventricular remodeling (95). As mentioned before, recovery of the heart after myocardial infarction is a complex process involving various inflammatory components and cardiomyocyte responses to ischemia (96). In this context, photobiomodulation, a non-invasive therapeutic modality that utilizes low-level light sources — typically low-power lasers or light-emitting diodes (LEDs) has proven to be a promising alternative (97). Oron's research team pioneered the demonstration of reduced mitochondrial damage and increased ATP content in the infarcted myocardial region of dogs treated with low-power lasers (96). The authors also demonstrated that low-power lasers reduced mortality and infarct size compared to untreated dogs. In addition to improving cellular energy potential, the mechanisms targeted by photobiomodulation to achieve a cardioprotective effect may include increased release of nitric oxide, vascular endothelial growth factor and new blood vessel formation (98, 99). Preclinical studies have also shown that photobiomodulation can modulate the enhanced inflammatory response after myocardial infarction. Our group has previously shown that application of low-power lasers to the myocardium immediately after coronary occlusion effectively reduces infarct size and the incidence of large infarcts and attenuates systolic dysfunction in rats at 3 days (100, 101). These findings were associated with reduced myocardial expression of IL-1β and IL-6 compared to non-irradiated rats. In two subsequent studies, our group carried out high-throughput gene expression analysis to identify differentially expressed genes in infarcted myocardium between 24 h and 3 days post-infarction, with low-power lasers therapy initiated approximately 60 s after coronary occlusion. Notably, low-power lasers induced a marked reduction in the mRNA expression of key mediators involved in post-MI inflammation and extracellular matrix remodeling, including IL-6, tumor necrosis factor receptor, transforming growth factor beta 1, and collagens type I and III (95, 102). Finally, additional studies utilizing prolonged low-power lasers therapy in infarcted rodent models have demonstrated improved outcomes in heart failure progression, including attenuation of myocardial hypertrophy and fibrosis, reduced pulmonary congestion, and enhanced left ventricular function (103, 104). These findings were associated with a potent antioxidant and anti-inflammatory effect of low-power lasers.

In summary, the immunomodulatory role of photobiomodulation, particularly low-power lasers therapy, may hold significant promise in attenuating the inflammatory response and promoting favorable post-infarction cardiac remodeling. This therapeutic strategy may be especially beneficial for individuals exhibiting an overactive and prolonged post-infarction inflammatory state, where improved inflammatory regulation could contribute to enhanced cardiac structure and function, reduced fibrosis, and decreased electrical instability via the suppression of pro-inflammatory cytokines (105–107).

## Treatment of hypertension, diabetes, and chronic kidney disease as a key inflammatory and neglected concomitant diseases

Together, several inflammatory pathways lead to atherosclerosis and its complications, but in addition to specific therapies, many drugs in clinical practice have anti-inflammatory effects. In this context, meta-analysis of inhibitors of the renin-angiotensin system showed a significant decrease in markers of inflammation (108). However, decrease in hsCRP obtained with a renin-angiotensin system blocker may be abolished by concomitant use of hydrochlorothiazide (109).

Several antidiabetic drugs have anti-inflammatory properties, such as pioglitazone (110), glucagon like peptide-1 receptor agonists (111), and dipeptidyl peptidase-4 inhibitors (112). Conversely, meta-analysis with 38 randomized controlled studies evaluated the effects of inflammatory markers among sodium-glucose cotransporter-2 inhibitors and did not find anti-inflammatory effects, including effects on hsCRP levels (113).

Lipid-lowering agents such as statins, present anti-inflammatory properties, reducing hsCRP and cardiovascular events (114, 115). The combination of statin with ezetimibe promoted an additional reduction on concentrations of hsCRP and in cardiovascular events when compared to statin monotherapy (116). Despite the benefits on cardiovascular parameters across hsCRP strata, the inhibitor of proprotein convertase subtilisin/kexin type 9 (PCSK9) evolocumab did not change C-reactive protein levels in the Fourier trial (117, 118). Inclisiran, a novel small-interfering RNA against PCSK9 did not show effects on markers of inflammation or adverse events in immune parameters (119).

In addition to the anti-inflammatory properties of renin-angiotensin system inhibitors, finerenone, a nonsteroidal selective mineralocorticoid antagonist presents anti-inflammatory effects, with proven renal and cardiovascular benefits (120–122). In the ischemia/reperfusion model of kidney injury, finerenone showed favorable effect increasing the M2 protective macrophages in glomeruli (122).

A healthy lifestyle also reduces inflammation markers. In fact, lower levels of hsCRP were observed among professional runners, despite their high-intensity training, and they showed lower intima-media thickness, and higher percentage of circulating endothelial progenitor cells (123, 124). In a large cohort, changes in lifestyle with better risk factor control were strongly and independently associated with lower hsCRP levels (125).

### Is there a link between inflammation and bleeding?

Acute coronary syndromes are related to increased bleeding risk after percutaneous coronary intervention compared to chronic coronary syndromes (126). In the JUPITER trial, among primary prevention patients with hsCRP levels ≥2 mg/L and relatively normal LDL-cholesterol levels, those treated with rosuvastatin had an impressive decrease in cardiovascular events (114). In the same trial a pre-specified secondary outcome was the effect of rosuvastatin in the rate of venous thromboembolism. Surprisingly, a marked decrease of venous thromboembolism was found among these patients, with elevated levels of hsCRP, receiving rosuvastatin (127). The link between C-reactive protein and thrombosis seems related to C-reactive protein destabilized isoforms that are not only pro-inflammatory but atherothrombotic (128).

The link between inflammation and bleeding was assessed in 1,864 consecutive patients with acute coronary syndromes. Patients were followed for one year, and baseline hsCRP levels were predictive of major cardiovascular outcomes, but not for bleeding risk (129). Therefore, inflammation *per se* does not seem related to bleeding, but possibly to increased thrombotic risk.

## Conclusions

In brief, addressing the residual inflammatory cardiovascular risk requires a comprehensive understanding of the intricate network of inflammatory pathways, whose relevance may vary between the acute and chronic phases of coronary artery disease. While lifestyle modifications and control of traditional risk factors remain fundamental, particularly in primary prevention, targeted modulation of inflammation, whether through specific cytokine inhibition or broader immunomodulatory approaches, holds significant promise, especially in the acute setting of myocardial infarction. Although biomarkers like hsCRP are valuable for risk stratification, they lack causal specificity. In contrast, IL-6 has emerged as a particularly promising therapeutic target, given its more direct mechanistic involvement in atherosclerotic inflammation. Furthermore, growing evidence supports the potential of leveraging immune-modulatory strategies, including vaccines, to achieve long-term reduction in cardiovascular events. Future research should focus on refining these interventions to balance efficacy and safety, ultimately translating into more personalized and effective cardiovascular care.
